# Incidence and challenges in management of hemodialysis catheter-related infections

**DOI:** 10.1038/s41598-022-23787-5

**Published:** 2022-11-29

**Authors:** Meriam Hajji, Manel Neji, Sahar Agrebi, Saoussen Ben Nessira, Fethi Ben Hamida, Samia Barbouch, Amel Harzallah, Ezzedine Abderrahim

**Affiliations:** 1 Department of Internal Medicine “A”, Charles Nicolle Hospital, Beb Saadoun, 1009 Tunis, Tunisie; 2 Faculty of Medicine of Tunis, University of Tunis El Manar, Tunis, Tunisia; 3 Laboratory of Renal Pathology (LR00SP01), Charles Nicolle Hospital, Tunis, Tunisia

**Keywords:** Health occupations, Nephrology

## Abstract

Catheter-related infections (CRI) are a major cause of morbidity and mortality in chronic hemodialysis (HD) patients. In this paper, we share our experience with CRI in HD patients. We recorded 49 cases of CRI among 167 patients during a period of 40 months (January 2018–April 2021). The incidence of CRI was 3.7 per 1000 catheter-days. The revealing symptoms were dominated by fever or chills (90%). Inflammatory signs were observed in 74% of cases with respectively concurrent exit-site (51%) and tunnel infection (6%). The biological inflammatory syndrome was found in 74% of patients (average CRP level = 198.9 mg/l). Blood cultures were performed in all cases and were positive in 65% of cases. Thirteen patients have been diagnosed with Infection complications, which were respectively infective endocarditis in 7 cases, septic arthritis in 3 cases, infective myositis in one case, cerebral thrombophlebitis in 1 case and mediastinitis in 1 case. The death occurred in eleven patients, it was due to septic shock in 9 cases, pulmonary embolism in one case and neurologic alterations related to cerebral thrombophlebitis. The mean seniority in HD was 16.5 months in the group with CRI and 3.7 months in the group without CRI (p < 0.04). We did not notice significant difference in mortality between tunnelled and non-tunnelled catheters. CRI does not seem to be more severe in patients with diabetes. Duration of use of the HD catheter (p < 0.007) and ferritin level (p < 0.0001) were independent factors that predispose to CRI in our population.

## Introduction

Catheter use in chronic haemodialysis patients has been recognized as distinct from other patient populations who require central venous access. According to the United States Renal Data System (USRDS) and the REIN register in France, respectively 80% and 57% of patients initiate haemodialysis (HD) via catheters^1,2^. Catheter-related infections (CRI) are common complications among patients on chronic HD and are associated with increased morbidity, hospitalization and death^3–8^. Actually, managing patients with CRI is really challenging, due to their need for regular hospital visits and constant interaction with several healthcare professionals.

In this paper we share the experience of a Tunisian center in managing HD patients and we report the characteristics and the risk factors of HD CRI in our patients. We also discuss the challenges of prophylactic strategies in this particular population.

## Patients and methods

We share the experience of the HD unit of the Department of Internal Medicine “A” at Charles Nicolle Hospital in Tunis during the period between the 2nd of January 2018 and the 30th of April 2021. We are in a university hospital and in our nephrology department, there is a dedicated HD unit including:

- A mini operating room for HD catheter placement.
- A pre-dialysis patient education program.
- A consultation office for regular follow-up and preparation for kidney transplantation.
- A day hospital for iron and antibiotics delivery and other nursing care.

We take care of 131 HD patients spread over three sessions in one day (from 7 a.m. to 7 p.m.), with an average rate of dialysis between two and three sessions per week for each patient. The medical staff includes three senior physicians with two resident physicians. The paramedical staff includes a chief nurse and twelve other nurses and two caregivers specialized in HD care.

### Study design

We prospectively collected data of all 160 patients for whom 167 HD catheters were placed in our department (6 patients had more than one HD catheter), including 49 patients who presented with CRI. Patients who had HD catheters in our department were either transferred to other HD centers or stayed in our HD unit until the agreement for reimbursement by the national health insurance funds. For these patients, we can therefore monitor them directly in-center hospital visit. For the other patients, we remote contact (phone) and monitoring; check-ups for the date of catheter removal, the cause of the removal (usable arteriovenous fistula or catheter related complications) became telephonic rather than face-to-face. In case if patients reported symptoms that can be related to CRI, there were calls or/and emails with the responsible dialyzer physicians for the respective centers in order to get information’s on the CRI type, treatment modalities and evolution. For patients with severe septic presentations, they were monitored in hospital.

We compared data and outcomes between two groups of patients (G1: with CRI) and (G2: non-CRI HD patients). For this, we used the ch^i2^test (or Fisher test if appropriate) to compare proportions. We calculated the incidence rates by relating the number of events to the duration of follow-up during the chosen period. In order to compare incidences in the two groups, we calculated the confidence intervals. The difference was considered significant if there is no overlap between the confidence intervals.

### Definitions

- Non-tunnelled catheter: is a central venous catheter placed percutaneously so that one end remains outside the body and the path between the skin and the vein is direct, it can be single or double lumen^9^.
- Tunnelled catheters: is a central venous catheter inserted in such a way that crosses subcutaneously over few centimeters and generally includes a cuff located just cephalad to the skin exit site. The cuff facilitates tissue ingrowth over a two- to three-week period, which anchors the catheter and minimizes bacterial migration from the exit site^9^.

The two types of tunnelled catheters used in the department were:

- Canaud catheter: which is a double catheter in silicone^10^.
- Palindrome catheter: which is a monobloc carbothane catheter, symmetrical, with a cuff^10^.

HD CRI have been defined based on the KDOQI 2019^11^:

- Catheter-related bloodstream infection (CRBSI): is the association of clinical manifestations with at least one positive blood culture with no other apparent source of infection and a positive culture for the same germ at the tip of the catheter^11^.
- Exit-site infection: Erythema or induration within 2 cm of the catheter exit site, in the absence of concomitant bloodstream infection (BSI) and without concomitant purulence^11^.
- Tunnel infection (TI) : Tenderness, erythema, or site induration 12 cm from the catheter site along the subcutaneous tract of a tunnelled catheter, in the absence of concomitant BSI^11^.
- Sepsis is a life-threatening organ dysfunction caused by an inappropriate host response to an infection^12^.
- Septic shock is the association of sepsis, need for vasoactive drugs to maintain mean blood pressure ≥ 65 mmHg and serum Lactates > 2 mmol/l despite adequate vascular filling^12^
- Infective endocarditis (IE): is a graft of an infectious agent, most often bacterial on a valvular or non-valvular endocardium, valvular prostheses or any other intracardiac prosthetic material. The endocardium is most often previously injured (subacute endocarditis or Osler endocarditis), or wholesome (acute endocarditis)^13^. IE diagnosis has been based on the DUKE criteria^14^.

### Statistical analysis

It was carried out through IBM SPSS statistics version 25.0 software. For the qualitative variables, the results were expressed in absolute and relative frequencies (%). Quantitative variables were represented by means, medians and standard deviations with determination of extreme values.

Several statistical tests were used to compare both qualitative and quantitative variables including t test, Chi-square (ϰ^2^), and if necessary, the exact test of Fisher and Mann–Whitney non parametric test according to the conditions required for their application.

An analytical study by the binary logistic regression method was carried out in order to identify the risk factors of CRI.

The survival data were studied by establishing the survival curves according to the Kaplan Meier method. The comparison of the survival curves was made by the Log Rank test.

Incidence rates were calculated by relating the number of events to the total duration of follow-up. Their confidence intervals were calculated at 95% by referring to the usual formulas.

In all statistical tests, the significance level was set at 0.05. Thus, there is a significant difference if p < 0.05.

### Ethical considerations

Informed consent was obtained from all subjects and/or their legal guardian(s). They have been informed that the material may show or include details of their medical condition or injury and any prognosis, treatment that they have, had or may have in the future, the article may be published in a journal which is distributed worldwide, and that their complete anonymity will be guaranteed.

### Ethical approval

All procedures in the present study were carried out in accordance with the institutional and national ethical guidelines for human studies and the guidelines proposed in the Declaration of Helsinki. The study was approved by the Ethics Committee of Charles Nicolle Hospital.

### Ethical statement

This material is the authors' own original work, which has not been previously published elsewhere. All authors have been personally and actively involved in substantial work leading to the paper, and will take public responsibility for its content.

## Results

**Figure 1:**
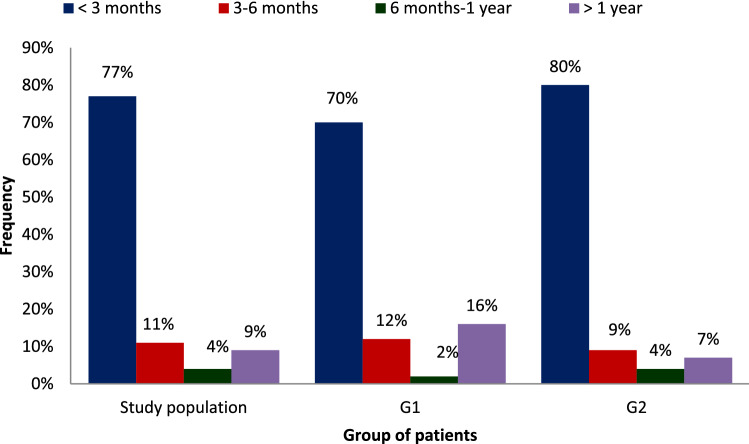
The distribution of our population according to seniority in hemodialysis.

During the study period, we recorded 160 patients for whom 167 HD catheters were placed in our department: 6 patients had more than one catheter including 49 patients presented with CRI. Baseline characteristics of our study population are illustrated in Table 1. The distribution of our population according to the seniority in HD was illustrated in Fig. 1.

**Table 1:** Patient characteristics according to the occurrence of CRI.

	Study population		Catheter related infection (+); number		Catheter related infection (−), number		P
	N	%	N	%	N	%	
N	160		49		111		
Mean age (range)	55.9 (17- 86)	–	58.2 (28–83)	–	54.9 (17–86)	–	NS
Gender ratio M/F	1.53	–	1.45	–	1.58	–	NS
Smoking intoxication	72	45	25	51	47	42	NS
Alcohol intake	7	4	1	2	6	5	NS
Drug addiction	3	1	–	–	3	3	NS
Hypertension	86	54	25	51	61	56	NS
Dyslipidemia	56	35	9	18	31	28	0.004
Diabetes	69	69	23	47	44	40	NS
History of infective endocarditis	2	1.3	0	–	2	2	NS
History of bacteraemia*	8	5	4	8	4	4	NS
History of CRBSI	2	1.3	2	4	0	0	NS
Initial nephropathy							NS
Diabetic nephropathy	64	40	21	43	43	39	
Vascular nephropathy	12	8	5	10	7	6	
CG nephropathy	12	8	2	4	10	9	
CTI nephropathy	15	9	2	4	13	12	
PKD	8	5	1	2	7	6	
ANCA vasculitis	7	4	3	6	4	4	
RPF	2	1	1	2	1	1	
undetermined nephropathy	39	24	14	29	25	22	
Chronic graft dysfunction	1	1	0	0	1	1	
BMI (kg/ m^2^) (extremes)	24.5 (18.9–34.6)	–	25.9	–	25.4	–	NS
BMI > 30 kg/m^2^	30	19	12	25	18	16	< 0.04
Poor personal hygiene status	35	22	27	55	8	6	< 0.001

*CRI* Catheter-related infection, *N* number, *not related to a CRI. *CRBSI* Catheter-related bloodstream infections, *CG* Chronic glomerular, *CTIN* Chronic tubulointerstitial, *PKD* Polycystic kidney disease, *RPF* Retroperitoneal fibrosis, *NS* non-significant.

**Figure 2:**
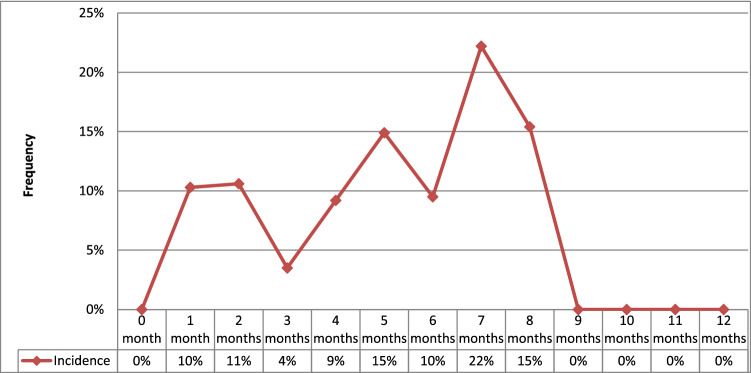
Monthly incidence of catheter related infection in our study population.

Seniority in HD compared to the date of the catheter insertion was 7.7 months ± 27.1 (0–240 months) in the overall population. It was 16.5 months (0–240 months) in G1 and 3.7 months (0–84 months) in G2 (p < 0.04). When to the rhythm of dialysis sessions, 134 patients were on dialysis at the rate of 3 sessions per week (84%), 47 of whom were in the G1 (96%, p < 0.04). One hundred and thirty-six catheters were placed for patients initiating HD (81.4%) (p = NS). Fistula thrombosis was the indication for HD catheter-placement in 15 cases (9%). Tunnelled Catheters were placed in 27% of patients (n = 45), of whom 35 were of the Canaud Vygon type and 10 were of the Palindrome type. Non-tunnelled Catheters use were predominant (n = 122, 73%) in the overall study population, mainly internal jugular Catheters (95%) with respectively (G1:86%) and (G2:98%). The difference was not statistically significant. The mean duration use of HD catheters, all types combined, was 65 days (extremes: 2–365 days). It was 70 days (extremes: 2–240 days) for non-tunnelled catheters and 102.4 days (extremes: 7–365 days) for tunnelled catheters, with respectively 67.1 days (extremes: 2–365 days) in G1 vs 42.8 days (2–365 days) in G2 (p < 0.04). After a cumulative follow-up duration of 457.7 months, 51 episodes of CRI were recorded in 49 patients corresponding to a monthly incidence of 11.1% (95% CI 8.08–14.0) or 3.7 per 1000 catheter-days (Fig. 2). The diagnostic delay, which is the median delay between HD catheter placement and the diagnosis of the CRI, was 65.1 days (extremes:2–365 days). Clinical manifestations of CRI included inflammatory signs (74%) with concurrent ESI (51%). Fever or chills were the most sensitive clinical features (90%) then tachycardia (12%) and hemodynamic instability (10%). TI was noted in 3 cases (6%).

C reactive protein (CRP) was positive in all G1 patients with an average rate of 198.9 mg/l (extremes: 57–899), hyperleukocytosis was noted in 38 cases (74%) with a mean white blood cell count of 12,750 per mm^3^ (extremes: 5210–30,460). Anaemia was noted in 83% of cases with a mean haemoglobin (Hb) rate of 8.7 g/dl (7 g/dl in G1 versus 9.5 g/dl in G2; p < 0.001). The mean of ferritninemia was 169.5 ng/ml in the population study, it was 683.5 ng/ml in G1 versus 150.3 ng/ml in G2 (p < 0.001). The average rate of albumin was 34.4 g/l, the difference between G1 and G2 and it was not significant.

**Figure 3:**
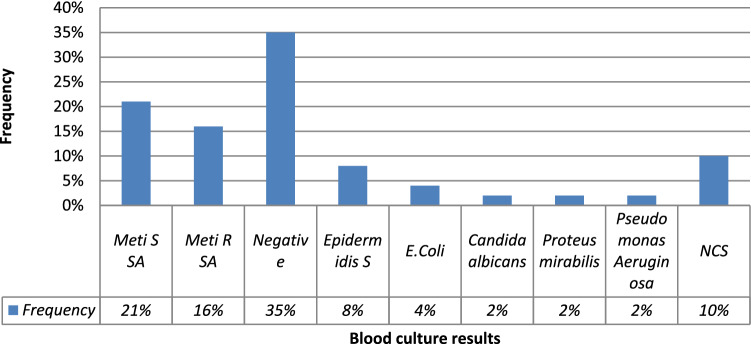
Blood cultures results in catheter related infection group.

Blood cultures were performed in all cases and were positive in 65% of cases (n = 33) (Fig. 3). The mean time between the catheter placement and CRI diagnosis was shorter with *Staphylococcus aureus* (*S. aureus*) than with the other germs: 36 days (extremes: 2–130 days) versus 97 days (extremes: 10–365 days). During the study period, among 49 patients with CRI, we reported 33 CRBSIs (65%), 25 ESI (51%) and 3 TI (6%). Table 2 summarizes CRI aspects respectively in tunnelled and non-tunnelled HD catheters.

**Table 2:** CRI according to the type of catheter.

	Non-tunnelled catheter (N = 122)	Tunnelled catheter (N = 45)	P
Catheter related infection (+); number	35	14	NS
Catheter related infection (−), number	85	31	
Use duration, mean (total); days	102.4 (8541)	70 (4363)	< 0.04
ESI (95% CI)	18 (0.26–5.32)	7 (1.1–3.9)	NS
TI	–	3	–
CRBSIs (95% CI)	24 (2.84–6.48)	9 (2.26–4.13)	< 0.05
Incidence CRI (95% CI); (/1000 catheter-days)	4.10 (2.74–5.46)	3.21 (1.53–4.89)	NS
CRI—staph meti R; number (incidence, 95% CI)	8 (0.94; [0.29–1.59])	2 (0.46; [0–0.64])	NS
CRI—other staph; number (incidence, 95% CI)	12 (1.4; [0.61–2.2])	0	< 0.05
CRI—GB (−); number (incidence, 95% CI)	4(0.47; [0.01–0.93])	1 (0.23; [0–0.68])	NS
CRI—HC (−); number (incidence, 95% CI)	6 (0.7; [0.14–1.26])	11(2.52; [1.03–4.01])	NS

*CRI* Catheter Related Infection, *CRBSIs* catheter blood stream infections, *ESI* exit site infections, *TI* tunnel infections, *Staph* Staphylococcus, *meti R* methicillin resistant, *GB* (−) gram negative bacillus, *HC* (−) negative blood culture, *N* number.

Thirteen patients have been diagnosed with Infection complications (26%). These were most common for *S. aureus* CRBSI, which were respectively: IE in 7 cases, septic arthritis in 3 cases, infective myositis in one case, cerebral thrombophlebitis in 1 case and mediastinitis in 1 case. IE was diagnosed based on transthoracic echocardiography that revealed a definite vegetation in mitral valve in 4 cases, aortic valve in 2 cases and tricuspid valve in 1 case.

All CRI patients were empirically treated with antibiotics to cover gram-positive organisms with a combination of intravenous (IV) Vancomycin (1 g twice per week post HD session) and IV Amikacin (500 mg) in 3 boluses divided according to the dialysis rhythm of each patient. The antibiotic regimen was adapted once culture and sensitivity results are available. For one patient who was diagnosed with candida albicans septicaemia, empirical antibiotic therapy was stopped and antifungal treatment with fluconazole was initiated with a loading dose of 800 mg on the first day then 400 mg per post dialysis day. For the one with positive blood cultures for *pseudomonas aeruginosa*, he had IV Imipenem at a dose of 1 g then 500 mg/day for 23 days in combination with Amikacin (500 mg) in post dialysis for a total of 3 boluses. A combination of ciprofloxacin at a dose of 200 mg/day for 14 days and IV Amikacin (500 mg) in 3 boluses according to the same regimen for one patient with positive blood culture for *proteus mirabilis.*

ESI were typically treated for 7 to 14 days and TIs, in the absence of a concurrent CRBSI, were treated for 14 days. Removal of HD catheters was indicated in 40 cases (78%): concomitantly with the diagnosis of CRI in 34 cases (30 of which were non-tunnelled catheters) and after an average delay of 5.5 days in 6 cases (15%). Indications of tunnelled Catheters removal were TI in 3 cases, including two cases where we identified *S. aureus,* one case where we identified *Pseudomonas aeruginosa,* CRBSI with blood cultures positive for *respectively candida albicans, E. coli *and *proteus mirabilis* respectively in one case each and septic shock in 2 patients with negative blood cultures.

**Figure 4:**
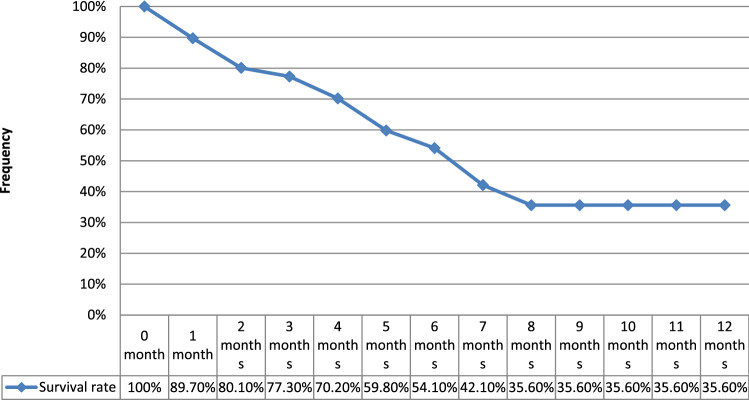
Survival rate of HD catheters without infection.

**Figure 5:**
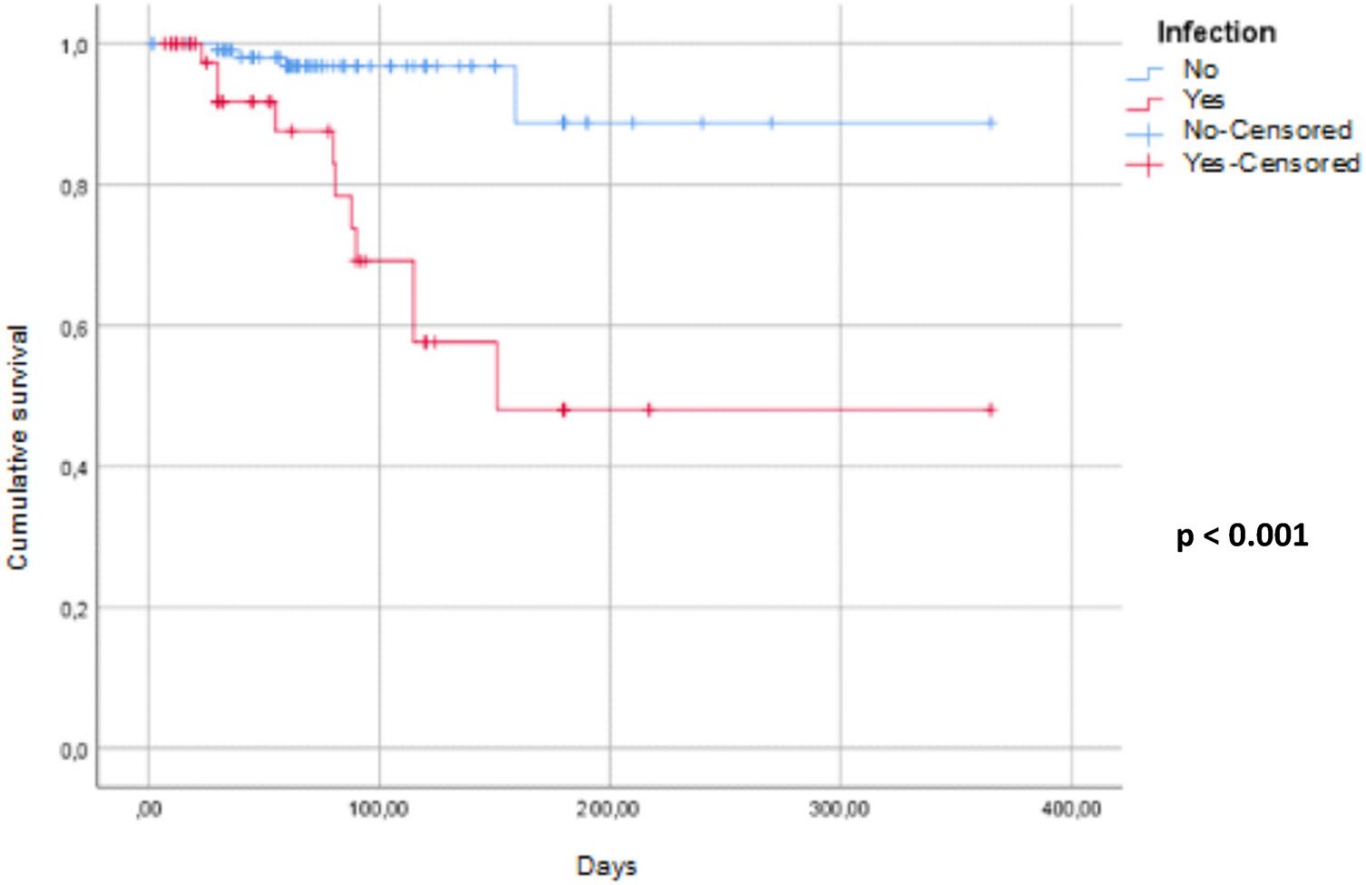
Comparative patients survival between groups with and without catheter related infections.

The monthly hospitalization rate was 9.53% (95% CI 6.61–12.44). The median hospitalization time of patients with CRI was 20.2 days (extremes: 1–45 days) with a monthly rate of 2.51 days per 100 patients (95% CI 2.35–2.65). Survival rate of HD catheters without infection is illustrated in Fig. 4. Overall monthly mortality was 3.49% (95% CI; 1.72–5.25). It was 10.36% (95% CI 4.24–16.48) in G1 vs 1.23% in G2 (95% CI 0.02–2.44). The specific monthly mortality related to CRI was 2.56% (95% CI 1.05–4.07). Seven deaths occurred in patients with methicillin-resistant *S. aureus* CRBSI (64%). Causes of death were septic shock in 9 cases, pulmonary embolism in one case and neurologic alterations related to cerebral thrombophlebitis in one case. Patients’ survival was represented in Fig. 5. Table 3 summarized the results of both univariate and multivariate analyses to identify factors influencing the occurrence of CRI.

**Table 3:** Risk factors of catheter-related infection.

Univariate analysis						
Parameters		CRI		RR	CI à 95%	P
		Yes	No			
Gender	Female	41	39	1	Reference	NS
	Male	59	61	1.09	[0.46–1.82]	
Age (years)		58.2	55	1.01*	[0.99–1.04]	NS
BMI	< 30 kg/m^2^	71	86	1	Reference	** < 0.04**
	> 30 kg/m^2^	29	14	2.37	[1.05–5.37]	
Poor personal hygiene status	No	71	14	1	Reference	** < 0.001**
	Yes	29	86	19.80	[7.65–51.32]	
Diabetes	No	53	59	1	Reference	NS
	Yes	47	41	0.52	[0.64–2.46]	
Seniority in HD (years)		16.5	3.7	1.02	[1.001–1.043]	** < 0.04**
History of bacteremia	No	88	96	1	Reference	NS
	Yes	12	4	2.38	[0.57–9.93]	
History of catheterization	No	84	95	1	Reference	** < 0.04**
	Yes	16	5	3.41	[1.12–10.45]	
Insertion site	Internal jugular	86	98	1	Reference	NS
	Femoral	10	0	3.60	[0.58–22.28]	
	Sub-clavian	4	2	2.40	[0.15–39.22]	
Type of catheter	Non-tunnelled	35	85	1	Reference	NS
	Tunnelled	14	31	1.19	[0.56–2.35]	
Number of haemodialysis sessions per week	< 3	4	78	1	Reference	** < 0.02**
	3	96	22	6.48	[1.47–28.64]	
Duration of the use of the catheter (months)		2.23	1.42	1.25**	[1.02–1.54]	** < 0.04**
Ferritinemia (ng/ml)		683.5	150.5	2.06***	[1.87–2.71]	** < 0.0001**
Hemoglobin (g/dl)		6.8	9.4	22.2	[7.9–62.5]	** < 0.0001**
Albumin (g/l)		37.1	36.7	1	[0.990–1.011]	NS

Multivariate analysis						
Parameters	RR	CI à 95%	P			
Poor personal hygiene status	29.09	[7.08–119.5]	< 0.0001			
Duration of use of the catheter(months)	1.51	[1.12–2.03]	< 0.007			
Ferritinemia (/100 ng/ml)	2.39	[1.63–3.51]	< 0.0001			

Significant values are in bold.

*BMI* Body mass index, *CRI* Catheter related infection.

*Per year. **Per month. ***Each increment of 100 ng/ml.

## Discussion

According to a large American meta-analysis, among 380,000 people requiring HD for ESRD in 2010, 18% of prevalent and 80% of incident dialysis patients used catheters for access^15^. Infection was the second leading cause of death among ESRD patients, and use of catheters as access was a predictor of all-cause and infection-specific mortality^15^. Hospitalizations for a vascular access infection are much higher with HD catheters than with AVF and polytetrafluoroethylene prostheses (PTFE)^16^. When using a catheter in HD, the risk of infection is multiplied by 3 compared to a prosthetic fistula and by 7 compared to a native AVF^17^. In our population, the incidence of CRI per 1000 catheter-days was 3.7. According to literature data, incidence rates of CRI vary widely, but within a rate of 0.46 to 30 per 1000 catheter-days^18–24^. We note that ours is almost in the lower limit, this could be explained by compliance with the rules of hygiene and asepsis and /or the limitation of the duration catheter placement (65 days: range: 2–365).

Regarding the cause of initial nephropathy, diabetes constitutes the leading cause of dialysis in developed countries^1,2,25,26^. In our study, clinical features of CRI were dominated by fever, chills such as in some studies^20,24^. In other studies, general signs such as nausea and vomiting were reported^27^. IE was the main infection complication in our study (14%) which agrees well with other studies’ results with a reported incidence of 3 to 17%^24,28,29^. The microbiologic profile of CRI varies over time and according to the studied populations. Several factors may be involved such as epidemiological variability, use of antibiotics, proportion of immunocompromised patients, etc. The most reported involved microorganisms in CRI are *Gram-positive cocci* and *Gram-negative bacilli (GNB)*^30–32^. *Enterococci* and *Candida* are less common^23–30^. The therapeutic approach depends on the catheter type, the isolated microorganism and the clinical manifestations at presentation. Empiric antibiotic treatment to cover gram-positive organisms is recommended till culture and sensitivity results are available^11^. The antibiotic choice Penicillin M or Cefazolin depends on the severity of the sepsis^33^. According to the American Society for Infectious Diseases, empiric antibiotic therapy should include vancomycin and cover *GNB* (3rd generation cephalosporin-carbapenem-β-lactamase)^34^. It also recommends switching from Vancomycin to Cefazolin in the case of methicillin susceptible *S. aureus.* In the case of a Vancomycin-resistant enterococcus, it is recommended to put on oral Daptomycin or Linezolid^34^. The recommended treatment duration remains controversial ranging from 3 weeks to only 14 days^35^. However, these recommendations were developed on non-HD patients^21,36^. According to the KDOQI, performing blood cultures is recommended before the start of empiric antibiotic therapy; the action to be taken will subsequently depend on the results of the microbiological samples^11^. The decision of catheter removal is sometimes ambiguous, especially for tunnelled catheters. Indications for catheter removal are non-tunnelled catheters, state of shock, infection of the catheter orifice with tunnel infection, persistent fever and positive blood cultures after 36 to 48 h of appropriate antibiotic therapy, recurrence despite appropriate antibiotic therapy, septic thrombosis authenticated by echo-Doppler, infective endocarditis or other secondary locations, after identification of certain germs: *S. aureus*, *Candida* and *Pseudomonas aeruginosa*^11,21,35,36^. Management of CRI in our study population, agreed well with these recommendations. Several factors of CRI were reported such as advanced age has also been described as a risk factor in several studies with a variable threshold^1,28,37^. Diabetes has been retained as a risk factor exposing to the occurrence of CRI by some studies^1,18,38^. The femoral site of catheter insertion has been identified as a risk factor in some studies^39–41^ unlike others^18,42,43^. A study carried out in Denmark showed that the risk of CRI was increased with non-tunnelled catheters than with tunnelled catheters^44^. Many studies have identified the duration of catheterization as an independent risk factor CRI^40,45–47^. Several studies have shown that a low Hb level represents a risk factor favouring CRI^27,29,48^. The DIALIN network report shows that an Hb level below 9 g/dl is a risk factor for catheter infection (p = 0.05)^38^. In our study, the univariate analysis found that the occurrence of CRI is related to the seniority in HD but this was not confirmed by the multivariate analysis as reported by Izoard et al.^17^. Other risk factors have been reported in the literature, such as hypoalbuminemia^27,42^, use of erythropoietin^27,48^, transfusion^27,38^. Infectious complications related to HD catheters have been considered by some authors to be among the most preventable nosocomial infections thanks to preventive measures^38^.

## Conclusion

Tunnelled or non-tunnelled catheter vascular access is often, an essential alternative to the proper management of chronic renal failure replacement therapy program in our country; in particular that the waiting deadlines for arteriovenous fistulas making are too long, because of the insufficiency of hospital departments of vascular surgery and the precariousness of the social conditions of our patients in order to access to the private health sector. On the other hand, the high rate of CRI in our study, leads us to more focus on preventive strategies on both sides of the nurse and the patient.

## Supplementary Information

Supplementary Information 1.

## Data Availability

The datasets generated and/or analysed during the current study are not publicly available, but are available from the corresponding author in a Supplementary Information file.
